# Quality of Life and Complications after Nipple- versus Skin-Sparing Mastectomy followed by Immediate Breast Reconstruction: A Systematic Review and Meta-Analysis

**DOI:** 10.1097/PRS.0000000000010155

**Published:** 2023-06-29

**Authors:** Marloes E. Clarijs, Noelle J. M. C. Vrancken Peeters, Sophie A. F. van Dongen, Linetta B. Koppert, Andrea L. Pusic, Marc A. M. Mureau, Bianca F. M. Rijken

**Affiliations:** Rotterdam, the Netherlandsand Boston, MA; From the Departments of 1 Surgical Oncology and Gastrointestinal Surgery; 4 Plastic and Reconstructive Surgery, Erasmus MC Cancer Institut, University Medical Center Rotterdam; 2 Erasmus University Rotterdam and Erasmus MC, University Medical Center Rotterdam; 3 Division of Plastic and Reconstructive Surgery, Patient Reported Outcomes, Value and Experience (PROVE) Center, Brigham and Women’s Hospital.

## Abstract

Nipple-sparing mastectomy (NSM) has emerged as an alternative procedure for skin-sparing mastectomy (SSM), followed by immediate breast reconstruction. Because oncologic safety appears similar, patient-reported outcomes (PROs) and complication risks may guide decision-making in individual patients. Therefore, the aim of this systematic review was to compare PROs and complication rates after NSM and SSM.

**Methods:**

A systematic literature review evaluating NSM versus SSM was performed using the Embase, MEDLINE, and Cochrane databases. Methodologic quality of the included studies was assessed using the Newcastle-Ottawa Quality Assessment Form for Cohort Studies. Primary outcomes were PROs and complications. Studies that evaluated BREAST-Q scores were used to perform meta-analyses on five BREAST-Q domains.

**Results:**

Thirteen comparative studies including 3895 patients were selected from 1202 articles found. Meta-analyses of the BREAST-Q domains showed a significant mean difference of 7.64 in the Sexual Well-being domain (*P* = 0.01) and 4.71 in the Psychosocial Well-being domain (*P* = 0.03), both in favor of NSM. Using the specifically designed questionnaires, no differences in overall satisfaction scores were found. There were no differences in overall complication rates between the two groups.

**Conclusions:**

Patient satisfaction scores were high after both NSM and SSM; however, NSM led to a higher sexual and psychosocial well-being. No differences in complication rates were found. In combination with other factors, such as oncologic treatments, complication risk profile, and fear of cancer recurrence, the decision for NSM or SSM has to be made on an individual basis and only if NSM is considered to be oncologically safe.

Breast cancer is one of the most commonly diagnosed female cancers, with an increasing incidence globally.^1^ An important part of curative treatment of breast cancer involves surgical removal of the tumor mass. In recent years, surgical treatment has been refined to improve aesthetic outcomes with comparable oncologic results. Although the trend has shifted toward breast-conserving therapy, including oncoplastic reconstruction techniques, a mastectomy with reconstruction still may be the best treatment in case of a high tumor breast volume ratio, absence of donor-site tissue, or fear of recurrence.^2^ Therefore, new surgical techniques for mastectomy, such as skin-sparing mastectomy (SSM) or nipple-sparing mastectomy (NSM), have been developed to improve appearances. With the identification of patients at high risk for breast cancer in case of *BRCA* mutation, the rate of prophylactic bilateral mastectomies and the desire for NSM has increased.^3^

NSM involves removal of all glandular breast tissue with preservation of the native skin envelope, inframammary fold, and nipple-areola complex (NAC), whereas SSM involves the removal of all glandular breast tissue and inframammary fold without NAC preservation.^4^ The NAC is a tremendously important component of the breast, considering the aesthetics and contribution to sexual pleasure. The main reason to preserve the NAC is for aesthetic purposes, and previous studies reported an improvement in patient satisfaction and psychological benefit after NSM.^4–6^ Although SSM can be followed by NAC reconstruction, the NAC is unique in its appearance and therefore difficult to reconstruct accurately. Outcomes of NAC reconstruction vary across previous studies.^7–9^

Despite an increasing number of women choosing NSM for aesthetic reasons, the oncologic safety of the procedure had been questioned. The main concern about preserving the NAC is exposing patients to a higher risk of occult NAC tumor involvement. Because the retroareolar tissue is not fully removed, more terminal duct lobular units could be left behind compared with SSM.^10,11^ Nevertheless, previous research has shown equal local recurrence rates and overall survival outcomes in carefully selected breast cancer patients after NSM compared with conventional mastectomies.^4,12–15^ The National Comprehensive Cancer Network suggested that NSM is feasible in patients with a tumor-areola distance of greater than 2 cm, no detection of cancer in the nipple, and early-stage breast cancer with favorable biological tumor characteristics.^16^ Although the oncologic safety was beyond the scope of this review, attention is needed for the selection criteria of NSM patients.

Given similar oncologic outcomes, NSM might offer better aesthetic results and higher patient satisfaction compared with SSM, suggesting that NSM might be superior to SSM,^17^ which can be measured using patient-reported outcomes (PROs). In contrast, NAC preservation may cause an increased risk of complications, such as necrosis, wound healing problems, infections (with implant loss), or nipple displacement, and this may negatively affect PROs. The aim of this systematic review was to evaluate PROs and complication risks in patients undergoing NSM versus patients undergoing SSM followed by immediate breast reconstruction.

## PATIENTS AND METHODS

### Literature Search

A systematic search was performed in Embase, MEDLINE, and Cochrane databases according to Preferred Reporting Items for Systematic Reviews and Meta-Analysis guidelines from inception to March 30, 2021. A search string was drafted with the help of an experienced librarian using search terms related to “nipple-sparing mastectomy” and “patient-reported outcomes.” The exact search syntaxes for the Embase, MEDLINE, and Cochrane databases are available. (**See Document, Supplemental Digital Content 1**, which shows the search syntaxes for the Embase, MEDLINE, and Cochrane databases, http://links.lww.com/PRS/F858.)

### Eligibility Criteria

Studies were included if they evaluated patient-reported outcomes after NSM with SSM as a control group. SSM was defined as removal of breast glandular tissue including excision of the NAC, with preservation of the skin envelope. NSM involved removal of the breast glandular tissue, preserving the skin envelope and a thin NAC flap. If an immediate reconstruction was performed, studies using total mastectomy and simple mastectomy with nipple reconstruction were considered to be the same procedure as a skin-sparing mastectomy. To facilitate comparison, only articles including quantitative PRO measurements (PROMs) were used. Both unilateral and bilateral procedures, and autologous or expander or implant-based breast reconstructions were included. Studies were excluded that (1) included male patients, (2) reported SSM without differentiating between skin-sparing or nipple-sparing surgery, (3) did not compare cohorts, and (4) described gender transition surgery.

### Study Selection

Two independent authors (M.C. and N.J.M.C.V.P.) initially reviewed all articles based on title and abstract. Discrepancies of inclusion were resolved by discussion in which an additional author was involved (B.F.M.R.) and consensus was found in all cases. Systematic reviews were excluded. An extensive analysis of the full-text was performed.

### Data Extraction

Data extraction from studies included the methodologic and baseline clinical aspects of the studies [eg, year of publication, study design, cohort selection, sample size, age of subjects, specific PROM, complications, and time from surgery to completion of questionnaires (follow-up)]. Mean scores and standard deviations for each BREAST-Q domain were abstracted. The domains included Satisfaction with Breasts, Physical Well-being, Psychosocial Well-being, Sexual Well-being, and Satisfaction with Outcome. Authors were contacted by e-mail to request unpublished data. Data collection was independently done by two researchers (N.J.M.C.V.P. and S.A.F.D.), and checked by a third researcher afterward (M.C.).

### Quality Assessment

Both authors (N.J.M.C.V.P. and S.A.F.D.) independently assessed the risk of bias in included studies using the Newcastle Ottawa Scale for nonrandomized studies. The articles were rated based on selection, comparability, and ascertainment of exposure or outcome of interest, resulting in a score from 0 to 8. The Newcastle Ottawa scale can be converted into Agency for Healthcare Research and Quality standards, which divides the quality of studies in good, fair, and poor quality.^18^ Disagreements were settled by consensus.

### Statistical Analysis

BREAST-Q data were pooled with random-effect meta-analyses to determine the mean differences and 95% confidence intervals. The *I*^2^ test was used to assess heterogeneity, expressed as the percentage of variability across studies. An *I*^2^ value greater than 50% was considered to represent significant heterogeneity. Weights were calculated based on the inverse variance method. BREAST-Q scores range from 0 to 100, with higher scores representing better satisfaction or well-being.^19^ RevMan software was used to calculate standard deviations from other related statistics, such as standard errors and confidence intervals, if articles did not report standard deviations. If required, the statistics of two groups (eg, younger/older age groups) were combined according to the formulas for combining summary statistics. The exact formulas are described in the Cochrane Handbook (chapter 6.5.2.1 and chapter 6.5.2.10).^20^ Funnel plots were created to display the risk of publication bias. All statistical analyses were conducted using RevMan (Version 5.4.1) and values of *P* ≤ 0.05 were considered statistically significant.^21^

## RESULTS

### Search Results

**Fig. 1.**
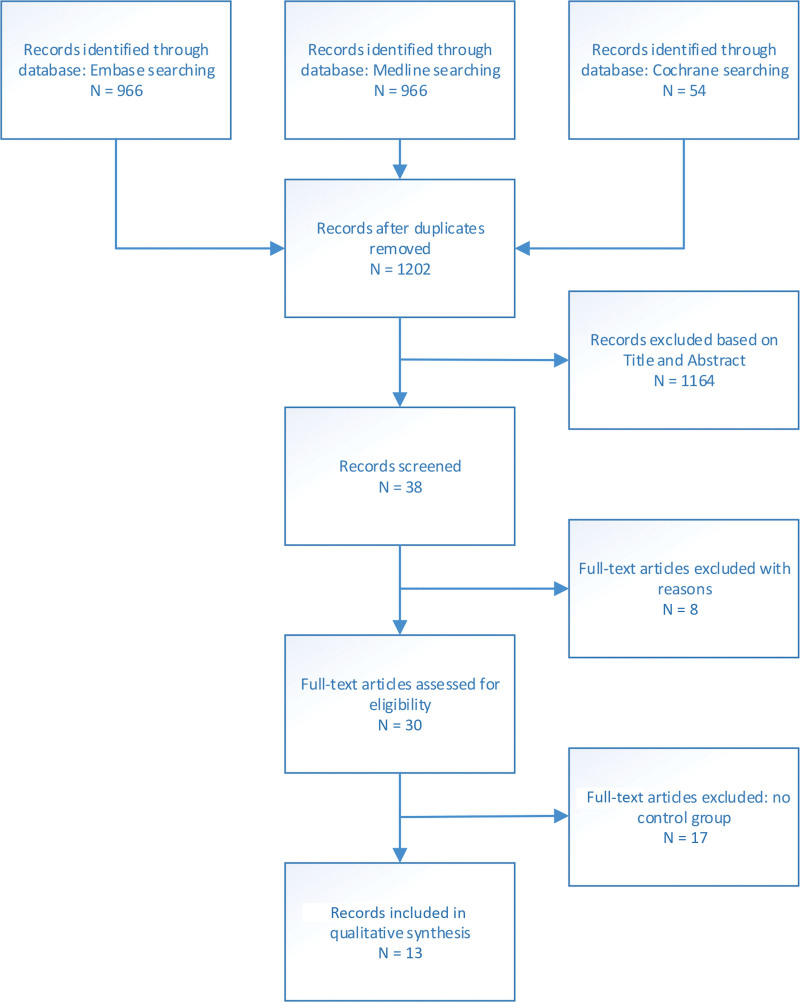
Flowchart study selection procedure.

After deduplication, a total of 1202 citations were identified. By screening title and abstract, 38 potentially relevant articles were selected, of which 16 articles were selected for full-text evaluation. Finally, 13 articles including 3895 patients met the criteria for inclusion in this systematic review (Fig. 1). The study-specific characteristics are summarized in Table 1.^5,6,17,22–31^

**Table 1.** Study Characteristics

Reference	Title	Study Type	Country	NSM	Control	Age (yr)	Outcome	Type of Reconstruction (%)	Bilateral Mastectomy (%)	Intraoperative or Adjuvant Radiotherapy (%)	Timing of Questionnaire after Surgery
Bailey et al., 2017^5^	Quality-of-Life Outcomes Improve with Nipple-Sparing Mastectomy and Breast Reconstruction	RO	United States	32	32	Mean: NSM, 48.9 SSM, 46.3	BREAST-Q	NSM: autologous (43.7) SSM: autologous (43.8) or expander-implant or direct-to-implant	NSM: 31 (96.6) SSM: 27 (84.4)	NSM: 5 (15.6) SSM: 7 (21.9)	>6 mo
Dossett et al., 2016^25^	Prospective Evaluation of Skin and Nipple-Areola Sensation and Patient Satisfaction after Nipple-Sparing Mastectomy	PO	United States	38	15	Mean: NSM, 49 SSM, 43	Breast Evaluation Questionnaire and Body Image after Breast Cancer Questionnaire	Immediate expander-implant (94) or autologous (6)	NSM: 33 (87) SSM: 15 (100)	NSM: 3 (8) SSM: 0	12 mo
Kim et al., 2019^29^	Comparative Study of Nipple-Areola Complex Position And Patient Satisfaction after Unilateral Mastectomy and Immediate Expander–Implant Reconstruction Nipple-Sparing Mastectomy versus Skin-Sparing Mastectomy	RO	Korea	55	85	Mean: NSM, 42.7 SSM, 45.6	Specifically designed questionnaire	Immediate expander-implant	Only unilateral procedures	NSM: 6 (10.9) SSM: 12 (14.1)	Unknown
Mesdag et al., 2017^6^	Nipple Sparing Mastectomy for Breast Cancer Is Associated with High Patient Satisfaction and Safe Oncological Outcomes	RO	France	63	89	Median: NSM, 49.5 SSM, 50	Specifically designed questionnaire	Immediate expander-implant (18.4) or direct-to-implant (81.6)	NR (mostly unilateral procedures)	NSM: 30.2% SSM: 10.1%	Median: 42 mo; IQR, 18–58 mo
Metcalfe et al., 2015^17^	Long-Term Psychosocial Functioning in Women with Bilateral Prophylactic Mastectomy: Does Preservation of the Nipple-Areola Complex Make a Difference?	CS	Canada	53	84	Mean: 41; range, 24–69	BREAST-Q IES HADS Decision Regret Scale	NR	Only bilateral procedures	NR	50 ± 31 mo
Opsomer et al., 2021^27^	Nipple Reconstruction in Autologous Breast Reconstruction after Areola-Sparing Mastectomy	RO	Belgium	55	348	Mean: 48.3	BREAST-Q	Autologous^a^	NSM: 22 (40) SSM: 83 (23.9)	NSM: 7 (12.7) SSM: 107 (30.7)	64.3 ± 18.9 mo
Ritter et al., 2021^23^	The Impact of Age on Patient-Reported Outcomes after Oncoplastic versus Conventional Breast Cancer Surgery	PO	Switzerland	32	31	NR	BREAST-Q	NSM: autologous (100)^b^ SSM: NR	NR	NR	35 ± 25 mo
Rojas et al., 2017^28^	The Impact of Mastectomy Type on the Female Sexual Function Index (FSFI), Satisfaction with Appearance, and the Reconstructed Breast’s Role in Intimacy	RO	United States	8	36	Median: NSM, 46.5 SSM, 50.5	FSFI	NR	NSM: 5 (62.5) SSM: 17 (42.2)	NSM: 0 SSM: 7 (19.4)	>1 yr
Romanoff et al., 2018^24^	A Comparison of Patient-Reported Outcomes after Nipple-Sparing Mastectomy and Conventional Mastectomy with Reconstruction.	RO	United States	219	1647	Mean: NSM, 44; TM, 48	BREAST-Q	Immediate expander-implant	NSM: 174 (79) TM: 1012 (61)	NSM: 9 (4) TM: 353 (21)	658 days; IQR, 442–1189 days
Santosa et al., 2019^22^	Comparing Nipple-Sparing Mastectomy to Secondary Nipple Reconstruction: A Multi-institutional Study	PO	United States	286	314	Mean: NSM, 44.0 SNR, 48.4	BREAST-Q	Immediate expander-implant or direct-to-implant	NSM: 216 (75.5) SNR: 184 (58.6)	NSM: 28 (9.8) SNR: 18 (5.7)	24 mo
Ueda et al., 2008^31^	Cosmetic Outcome and Patient Satisfaction after Skin-Sparing Mastectomy for Breast Cancer with Immediate Reconstruction of the Breast	PO	Japan	33	41	Mean: NSM, 44 SSM, 47	Specifically designed questionnaire	NSM: autologous (97) (one implant) SSM: autologous (100)^c^	NR	NSM: 1 (3) SSM: 1 (2)	50 mo NSM: 53 mo SSM: 47 mo
van Verschuer et al., 2016^30^	Patient Satisfaction and Nipple-Areola Sensitivity after Bilateral Prophylactic Mastectomy and Immediate Implant Breast Reconstruction in a High Breast Cancer Risk Population	PO	The Netherlands	20	25	Median: NSM, 37 SSM, 34	BREAST-Q	Immediate expander-implant	Only bilateral procedures	NSM: 2 (10) SSM (0)	Median NSM: 27 mo; range, 10–58 mo SSM: 65 mo; range, 43–136 mo
Wei et al., 2016^26^	Psychosocial and Sexual Well-Being following Nipple-Sparing Mastectomy and Reconstruction	PO	United States	52	202	Mean: NSM, 44.9 SSM, 45.7	BREAST-Q	Immediate expander-implant	NSM: 37 (71) SSM: 132 (65)	NSM: 6 (11.8) SSM: 15 (7.4)	NSM: 18.3 ± 17 SSM: 32.9 ± 21

RO, retrospective cohort study; PO, prospective cohort study; CS, cross-sectional survey study; IES, Impact of Event Scale; HADS, Hospital Anxiety and Depression Scale; FSFI, Female Sexual Function Index; TM, total mastectomy; SNR, simple mastectomy with nipple reconstruction; NR, not reported; DIEP, deep inferior epigastric perforator; IQR, interquartile range.

a DIEP flap, superior gluteal artery perforator flap, or lumbar artery perforator flap.

b DIEP flap.

c DIEP flap, latissimus dorsi myocutaneous flap, or transverse rectus abdominis myocutaneous flap.

### Study Characteristics

The intervention of interest was NSM, the comparator was SSM, always followed by immediate breast reconstruction. In three studies, a total mastectomy or “simple mastectomy with nipple reconstruction” was used as the control group.^22–24^ Seven articles were retrospective cohort studies, whereas six were prospective cohort studies. There was one cross-sectional survey study.^17^ The studies were conducted in the United States (*n* = 6), Europe (*n* = 4), Canada (*n* = 1), and Asia (*n* = 2). Studies included women diagnosed with invasive breast cancer or ductal carcinoma in situ. Four studies included both breast cancer patients and patients with a *BRCA* mutation opting for prophylactic mastectomy.^22,24–26^ Seven studies included both unilateral and bilateral mastectomies,^5,22,24–28^ one study only unilateral,^29^ and two studies only bilateral (prophylactic) mastectomies.^17,30^ Three studies did not specify this, resulting in at least 1436 unilateral and 2170 bilateral mastectomies. Six studies evaluated expander or implant-based reconstructions.^6,22,24,26,29,30^ Three studies included both expander or implant-based and autologous,^5,23,25^ and two studies, only autologous breast reconstructions.^27,31^ Sample sizes ranged from 44 to 1866 patients, with a mean of 73 patients undergoing NSM and 227 patients undergoing SSM. Eight studies used the BREAST-Q of which different domains were selected, five studies used another questionnaire. Studies had a mean follow-up time starting from 6 months after surgery. Nipple reconstruction after SSM was reported in seven studies, of which four studies specified the type of nipple reconstruction after SSM (eg, intradermal tattooing, local transposition flaps).^6,22,24,26,27,29,30^

### Quality Assessment

Six studies were rated as good, eight studies were rated as fair, and one study was rated as poor. Adjustment for potential confounding was not consistent across studies; seven studies adjusted for age, four adjusted for prognostic factors such as tumor grade and treatment, whereas other studies adjusted for income or insurance status (Table 2). The funnel plots did not indicate a risk of publication bias. (**See Figure, Supplemental Digital Content 2**, which shows funnel plots created to display the risk of publication bias for each domain of the BREAST-Q, http://links.lww.com/PRS/F859.)

**Table 2.** Quality Assessment of Nonrandomized Studies Based on the NOS and AHRQ^a^

Reference	Selection				Comparability	Outcome			NOS	AHRQ
	Representativeness of the Exposed Cohort	Selection of the Nonexposed Cohort	Ascertainment of Exposure	Outcome of Interest Was Not Present at Start of Study	Comparability of Cohorts on the Basis of the Design or Analysis	Assessment of Outcome^b^	Follow-Up Long Enough for Outcomes to Occur	Adequacy of Follow-Up of Cohorts	Total Score	
Bailey et al., 2017^5^	1	1	1	1	1	—	0	1	6	Fair
Dosset et al., 2016^25^	1	1	1	1	0	—	1	1	6	Fair
Kim et al., 2019^29^	1	1	1	1	2	—	0	0	6	Fair
Mesdag et al., 2017^6^	1	1	1	1	0	—	1	0	5	Fair
Metcalfe et al., 2015^17^	1	1	1	1	2	—	1	1	8	Good
Opsomer et al., 2020^27^	1	1	1	1	0	—	1	0	4	Poor
Ritter et al., 2021^23^	1	1	1	1	1	—	1	1	6	Fair
Rojas et al., 2017^28^	1	1	1	1	1	—	1	0	5	Fair
Romanoff et al., 2018^24^	1	1	1	1	2	—	1	1	8	Good
Santosa et al., 2019^22^	1	1	1	1	2	—	1	0	7	Good
Ueda et al., 2008^31^	1	1	1	1	0	—	1	0	5	Fair
van Verschuer et al., 2016^30^	1	1	1	1	0	—	1	1	6	Fair
Wei et al., 2016^26^	1	1	1	1	2	—	1	1	7	Good

NOS Score, Newcastle-Ottawa Scale; AHRQ, Agency for Healthcare Research and Quality.

a Maximum score is 8 (selection = 4, comparability = 2, and outcome = 2).

b Patient-reported outcomes are self-reported.

### Meta-Analyses of Mean BREAST-Q Scores

**Fig. 2.**
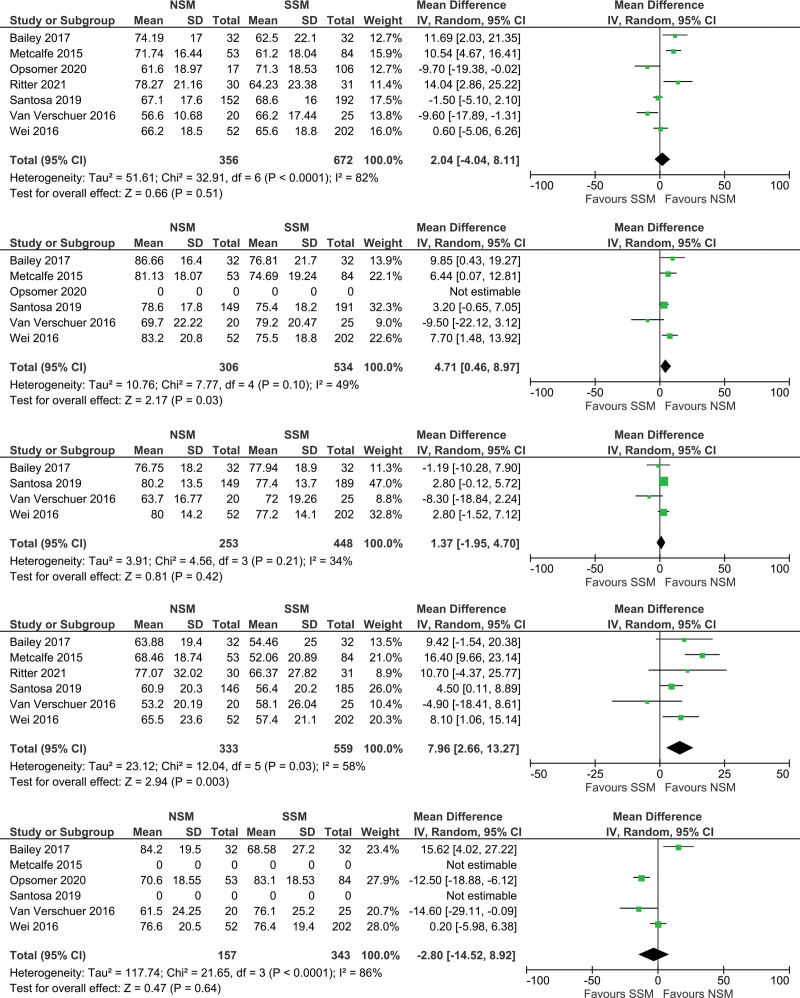
Forest plot showing the mean differences for the Satisfaction with Breasts, Psychosocial Well-being, Physical Well-being, Sexual Well-being, and Satisfaction with Outcome domains of the BREAST-Q.

Seven studies were included in the meta-analyses to evaluate mean BREAST-Q scores.^5,17,22,23,26,27,30^ A meta-analysis was performed for each domain: Satisfaction with Breasts, Psychosocial Well-being, Physical Well-being, Sexual Well-being, and Satisfaction with Outcome (Fig. 2). There was a significant mean difference of 7.96 in Sexual Well-being (*P* = 0.003) and 4.77 in Psychosocial Well-being (*P* = 0.01), both in favor of NSM. No statistically significant differences between NSM and SSM in Satisfaction with Breasts (2.04; *P* = 0.51), Satisfaction with Outcome (2.80; *P* = 0.64), and Physical Well-being (1.37; *P* = 0.42) were found.

Although Romanoff et al.^24^ evaluated BREAST-Q scores of NSM and total mastectomy patients, they were unable to share their data. Therefore, the data are missing in the meta-analyses. Their results showed that, after adjusting for relevant clinical variables (eg, age, unilateral versus bilateral mastectomy, chemotherapy, radiotherapy, and baseline BREAST-Q score), only Psychosocial and Physical Well-being were significantly higher in the NSM group.

### Other PROs

Besides the BREAST-Q, other specifically designed questionnaires were used to study quality of life as well (eg, harmony between breasts and sexual well-being). Ueda et al.^31^ showed that according to the Quality of Life Questionnaire for Cancer Patients Treated with Anticancer Drugs: the Breast response, the mean scores for patient satisfaction did not differ between SSM and NSM and were similar to those of patients undergoing breast-conserving therapy. Mesdag et al.^6^ used their own specifically designed questionnaire that was partially adapted from the BREAST-Q, as specific questions about nipple sensitivity or harmony between breasts were added. Most patients in the total cohort [NSM (*n* = 41), SSM (*n* = 63), or SSM with nipple reconstruction (*n* = 35)] were very satisfied with the overall aspect of the reconstructed breast [*n* = 73 (76.8%)], and there were no differences in satisfaction rates between the three groups. Focusing on the aesthetic results of the NAC, patient satisfaction with NSM was comparable to that of patients with secondary NAC reconstruction [NSM, *n* = 27 (75%) versus SSM, *n* = 18 (60%); *P* = 0.202]. Considering harmony between breasts, patients in the NSM group had a lower satisfaction rate compared with patients with SSM and nipple reconstruction [NSM, *n* = 14 (37.8%) versus SSM, *n* = 19 (63.3%); *P* = 0.038]. Rojas et al.^28^ reported that there were no significant differences in postoperative total median Female Sexual Function Index scores between the groups. Interestingly, women who underwent SSM experienced a significantly higher sexual satisfaction score than women who underwent NSM (median, 5.2 versus 4.8; *P* = 0.005). Kim et al.^29^ used a NAC-specific questionnaire consisting of nine items in which each item was scored on a five-point Likert scale. Their study showed a similar satisfaction about breast reconstruction between the NSM and SSM groups (3.48 for the NSM group versus 3.51 for the SSM group; *P* = 0.913). In addition, the NAC sensitivity score was significantly better in the NSM group compared with the SSM group (2.12 versus 1.84, respectively; *P* = 0.003). NAC position was better in the SSM group (2.88 in the NSM group compared with 3.80 in the SSM group; *P* = 0.001). Van Verschuer et al.^30^ found almost total loss of sensitivity in the NSM group, which was significantly lower compared with nonoperated women, measured with Semmes-Weinstein monofilaments (mean, 1.9 versus 4.7; *P* < 0.01). Dosset et al.^25^ used a Breast Evaluation Questionnaire and Body Image after Breast Cancer Questionnaire to evaluate quality of life. Similar to Kim et al.,^29^ they found that patients who underwent SSM were more satisfied with nipple position (*P* = 0.03). There was significant loss in (monofilament) sensation following mastectomy compared with preoperative sensation.

### Complications

Complication rates were reported in eight studies (Table 3). One study found more delayed wound healing in NSM patients [18 of 55 (32.7%) versus 15 of 85 (17.6%); *P* = 0.04], including postoperative NAC necrosis, but total reconstruction failure was more common in SSM patients [six if 85 (7.1%) versus zero of 55; *p* = 0.043].^29^ One study reported more overall complications in the NSM group, including primarily minor infections and minimal mastectomy flap necrosis (*P* = 0.046).^24^ One study showed a similar 2-year complication [76 of 286 (26.6%) versus 69 of 314 (22.0%); *P* = 0.73] and reconstruction failure rate [19 of 286 (6.6%) versus nine of 314 (2.9%); *P* = 0.90] between NSM and SSM.^22^ The other five studies could also not find a significant difference in the overall complication rates between NSM and SSM.^5,6,22,26,30^

**Table 3.** Comparison of Complications between NSM and SSM

Reference	Type of Complication(s)	SSM (%)	NSM (%)	*P*
Bailey et al. 2017^5^	No significant difference in any complication rates			NR
	Tissue expander infection	4 (7.1)	6 (10.3)	0.74
	Delayed wound healing complications	1 (1.8)	2 (3.4)	1.0
Kim et al. 2019^29^	Tissue expander-associated complications	27 (31.8)	21 (38.2)	0.435
	Delayed wound healing complications	15 (17.6)	18 (32.7)	0.04^a^
	Expander removal	5 (5.9)	0	0.067
	Implant associated complications	10 (12.5)	10 (18.2)	0.361
	Final reconstruction failure	6 (7.1)	0	0.043^a^
Mesdag et al., 2017^6^	No significant difference in overall complications	16 (18)	14 (22.2)	0.52
	Rate of skin-flap necrosis	6 (6.7)	0	0.042^a^
	Prosthesis contracture rate	2 (2.2)	7 (11.1)	0.034^a^
	NAC necrosis		4 (6.4)	NR
	Reconstruction failure	8 (9)	2 (3.2)	0.20
Opsomer et al. 2021^27^	Wound problems at nipple level	11 (3.2)	6 (10.9)	0.013^a^
	Necrosis of the nipple§	22 (6.3)	9 (16.4)	NR
Romanoff et al., 2018^24^	Overall complications			0.046^a^
	None	1444 (88)	179 (82)	
	Minor	160 (10)	32 (15)	
	Major	43 (3)	8 (4)	
Santosa et al., 2019^22^	No significant difference in overall complications	69 (22)	76 (26.6)	0.079
	Mastectomy flap necrosis	15 (4.8)	25 (8.7)	0.024^a^
	Reconstructive failure	9 (2.9)	19 (6.6)	0.081
Van Verschuer et al., 2016^30^	No significant difference in overall complications	9 (38)	12 (60)	0.14
	NAC necrosis^b^	2 (8)	0	NR
	Mastectomy skin flap necrosis	0	1 (5)	NR
Wei et al., 2016^26^	No significant difference in overall complications			0.207
	None	180 (89.1)	43 (82.7)	
	≥1 complication	22 (10.9)	9 (17.3)	

NR, not reported.

a *P* < 0.05.

b Spared and reconstructed nipple.

## DISCUSSION

The aim of this study was to conduct a systematic review and meta-analysis to compare PROs and complication rates between NSM and SSM. Statistically significant differences in favor of NSM were found in the Psychosocial and Sexual Well-being domains of the BREAST-Q. The studies that analyzed PROMs other than the BREAST-Q found no differences in overall satisfaction scores. In general, nipple sensitivity was better, whereas nipple position was worse in NSM patients. Most studies did not show significant differences in overall complication rates; however, wound-related complications and NAC necrosis were more prevalent in NSM patients.^22,24,27,32^

A previously published systematic review that mainly focused on oncologic outcomes after NSM and SSM did not find any differences in local recurrence rates at 5 years or disease-free survival.^4^ Although aesthetic outcomes, PROs, and quality of life were evaluated as well, no scientific conclusions could be drawn because of the lack of standardized assessment tools. We used outcomes of the well-validated BREAST-Q that may have allowed for more reliable comparisons in our meta-analyses. Other modified questionnaires may not be specific enough to detect small differences in quality of life between surgical techniques. Such detailed aspects of psychosocial or physical well-being can be highly relevant to evaluate outcomes after breast reconstructive surgery, and this requires a reliable and validated questionnaire, for which the BREAST-Q has become the standard.^19^

An interesting finding was that a higher satisfaction of the nipple position was found in the SSM group. A possible explanation is that the position of the nipple can be adjusted to the new aesthetic contour of the breast during nipple reconstruction, whereas after NAC preservation, the position of the nipple is more determined within the skin flap. Moreover, use of a tissue expander may deteriorate the position of the NAC during expansion—specifically, in ptotic breasts. In exceptional cases, expanding the skin with or without the pectoral muscle results in an unacceptable position of the NAC that has to be corrected by NAC removal and reconstruction. Consequently, this might have a substantial impact on patient well-being. In patients with ptotic breasts, the new NAC position is even more difficult to predict after tissue expansion and therefore NSM is not always an option.^33–35^ Satisfaction with nipple position may also depend on whether the procedure was performed unilaterally or bilaterally, as in prophylactic mastectomies in *BRCA* mutation carriers.^36^ In the study by van Verschuer et al., a trend toward higher satisfaction with nipple position was seen in patients with bilateral SSM and nipple reconstruction.^30^ Strictly, unilateral and bilateral mastectomies should be analyzed separately to obtain more reliable outcomes.

Postoperative NAC sensitivity was investigated in three studies using different measurements. Two studies used an objective method with Semmes-Weinstein monofilaments, and both found almost no NAC sensation in the NSM group compared with nonoperated controls or preoperative sensation.^25,30^ In contrast, the third study found a higher NAC sensitivity score in the NSM group versus the SSM group based on a questionnaire.^29^ Previous literature describes the influence of incision type on postoperative sensation, with periareolar incisions causing more sensation loss compared with inframammary incision.^37^ Remarkably, van Verschuer et al. and Dosset et al. used different incisions, and both found substantial loss of sensation.^25,30^ For some patients, nipple reconstruction after SSM is an important final step to complete the aesthetic aspect of the breast and may improve PROs.^7,38^ Although some articles provided a detailed description of the procedures for nipple reconstruction, the timing of reconstruction was not reported.^6,17,24,26,27,29,30^ In general, nipple reconstruction is performed at least 3 months after surgery and includes local transposition flaps, intradermal tattooing, or a combination of both. Because PROs were collected starting from 6 months after surgery, we assumed that the nipple reconstruction had been completed before administration of the questionnaires.

This review included articles based on different kinds of reconstructions [eg, autologous or expander or implant-based reconstruction], which may have led to a heterogeneous sample. Although this may have influenced PROs,^39,40^ there was not sufficient data for a subgroup meta-analysis. Moreover, outcomes of the individual articles did not show a trend based on type of reconstruction.

Radiation therapy can have adverse effects on the skin or breast implant, but the influence of adjuvant radiotherapy on aesthetic outcomes and satisfaction was poorly documented. One study showed that a higher number of patients received adjuvant radiotherapy in the NSM group compared with the SSM group, causing more capsular contractures and therefore resulted in a lower satisfaction with harmony between breasts. Despite this finding, reconstruction failure was similar between NSM and SSM patients. Moreover, an association between radiotherapy and risk of NAC necrosis was not found.^6^ According to the BREAST-Q scores, one study revealed adjuvant radiotherapy as a significant negative predictor for the Satisfaction with Breasts, Sexual Well-being, and Satisfaction with Outcome domains.^24^ In contrast, Wei et al. did not find an association between radiotherapy and the Sexual Well-being domain.^26^

### Strengths and Limitations

One of the key strengths of the present review is the evaluation of validated BREAST-Q scores that allowed us to perform several meta-analyses. Heterogeneity in PROMs limited such comparisons in a previous study.^4^ Most of the included articles were of good quality. Besides PROs, a clear overview of the complications was also provided, as they may influence PROs. Unfortunately, there was some selective reporting bias in one article.^24^ Summary data for the mean preoperative BREAST-Q scores was missing and not available on request. Despite the fact that their statistical data could not be included in our meta-analyses, they found similar results in the BREAST-Q domains. Second, the follow-up for completion of the questionnaires varied between studies from 6 months up to 11 years. Previous research has shown that a longer follow-up results in improved quality-of-life outcomes.^24,41^ Therefore, results of our meta-analyses must be viewed with caution. Another limitation was the nonrandomized design of all studies. Thus, it is likely that patients were not eligible for both types of breast surgery and baseline characteristics may have differed. This could have led to confounding by indication (eg, if patients had ptotic or nonptotic breasts and underwent unilateral or bilateral procedures). The amount of skin preserved in SSM patients was unclear because of different provided definitions of SSM (eg, total, non–nipple-sparing, or simple mastectomies with implant-based reconstructions), which may have had a small effect on the outcomes, and this should be taken into account when interpreting the findings. The BREAST-Q is able to detect differences between preoperative and postoperative scores, but the cross-sectional or retrospective design of the studies prohibited such comparisons.

## CONCLUSIONS

Our meta-analyses showed significant differences in the Psychosocial Well-being and Sexual Well-being domains of the BREAST-Q in favor of NSM. Although complication types varied between NSM and SSM, there was no significant difference in the overall complication rate or reconstruction failure. For patients with an indication for mastectomy with immediate reconstruction who value the preservation of their NAC, NSM could be seen as a superior treatment. NSM should therefore be offered to selected patients with an indication or wish for mastectomy with immediate reconstruction, and in whom NSM is oncologically safe.

## DISCLOSURE

Dr. Pusic is a co-developer of the BREAST-Q (owned by Memorial Sloan-Kettering Cancer Center) and may receive royalties when it is used in for-profit, industry-sponsored clinical trials. The remaining authors have no financial interest to declare in relation to the content of this article. This research received no external funding.

## ACKNOWLEDGMENT

The authors wish to thank Sabrina T. G. Meertens-Gunput (Biomedical Information Specialist) from the Erasmus MC Medical Library for developing and updating the search strategies.

## Supplementary Material
