# Supplementary material for: Quality of Life and Complications after Nipple- versus Skin-Sparing Mastectomy followed by Immediate Breast Reconstruction: A Systematic Review and Meta-Analysis
Source: Plast Reconstr Surg. 2023 Jun 29;152(1):12–24. doi: 10.1097/PRS.0000000000010155 (PMC10298179; doi:10.1097/PRS.0000000000010155)
Supplement: Supplementary file 1 [file prs-152-012e-s001.pdf]

Supplemental Digital Content 1. Text - Search strategy

The search syntaxes for the Embase, Medline, and Cochrane databases.

Embase

('subcutaneous mastectomy'/de OR 'subcutaneous nipple sparing mastectomy'/de OR 'nipple sparing mastectomy'/de OR 'areola sparing mastectomy'/de OR ((nipple/de OR 'breast areola'/de) AND mastectomy/de AND preservation/de) OR (((subcutan*) NEAR/3 (mastectom* OR breast-amputat* OR breast-resect* OR mammectom*)) OR ((nipple OR areola) NEAR/3 (sparing OR preserv* OR retain* OR retention* OR conservation* OR conserving OR graft*) AND (mastectom* OR breast-amputat* OR breast-resect* OR mammectom*))):ab,ti,kw) AND ('treatment outcome'/de OR 'patient-reported outcome'/de OR 'cancer recurrence'/de OR 'cancer free survival'/de OR 'cancer survival'/de OR 'cancer mortality'/de OR mortality/de OR 'survival'/de OR reoperation/de OR 'complication'/de OR 'postoperative complication'/de OR 'necrosis'/de OR 'breast necrosis'/de OR 'surgical infection'/de OR 'infectious complication'/de OR 'hematoma'/de OR 'seroma'/de OR 'epidermolysis'/de OR 'prosthesis complication'/de OR 'prosthesis loosening'/de OR 'cancer specific survival'/de OR 'disease free survival'/de OR 'overall survival'/de OR 'skin necrosis'/de OR (outcome* OR recorren* OR disease-free OR cancer-free OR surviv* OR mortalit* OR reoperation* OR complication* OR necrosis* OR necrotic* OR hematoma* OR haematoma* OR epidermolys* OR seroma* OR (Prosthesis NEAR/3 (loss)) OR ((surgical OR wound) NEAR/3 infection*) OR ((oncological) NEAR/3 (safety))):ab,ti,kw) NOT ('sex reassignment'/mj OR transsexualism/mj OR 'transgender'/mj OR 'male breast cancer'/mj OR (sex-reassignment* OR transsexual* OR transgender* OR male-breast-cancer):ti) NOT ('case report'/de OR (case-report*):ti) NOT ([animals]/lim NOT [humans]/lim) NOT [conference abstract]/lim AND [english]/lim

Medline

(Mastectomy, Subcutaneous/ OR ((Nipples/) AND Mastectomy/ AND Preservation, Biological/) OR (((subcutan*) ADJ3 (mastectom* OR breast-amputat* OR breast-resect* OR mammectom*)) OR ((nipple OR areola) ADJ3 (sparing OR preserv* OR retain* OR retention* OR conservation* OR conserving OR graft*) AND (mastectom* OR breast-amputat* OR breast-resect* OR mammectom*))).ab,ti,kf.) AND (Treatment Outcome/ OR Patient Reported Outcome Measures/ OR Recurrence / OR Mortality/ OR Survival/ OR Reoperation/ OR complication.fs. OR Postoperative Complications/ OR Necrosis/ OR Surgical Wound Infection/ OR Hematoma/ OR Seroma/ OR Prosthesis Failure/ OR Disease-Free Survival/ OR (outcome* OR recorren* OR disease-free OR cancer-free OR surviv* OR mortalit* OR reoperation* OR complication* OR necrosis* OR necrotic* OR hematoma* OR haematoma* OR epidermolys* OR seroma* OR (Prosthesis ADJ3 (loss)) OR ((surgical OR wound) ADJ3 infection*) OR ((oncological) ADJ3 (safety))).ab,ti,kf.) NOT (exp *Sex Reassignment Procedures/ OR * Transsexualism/ OR * Transgender Persons/ OR * Breast Neoplasms, Male/ OR (sex-reassignment* OR transsexual* OR transgender* OR male-breast-cancer).ti.) NOT (case reports/ OR (case-report*).ti.) NOT (exp animals/ NOT humans/) AND english.la.

Cochrane

((((subcutan*) NEAR/3 (mastectom* OR breast-amputat* OR breast-resect* OR mammectom*)) OR ((nipple OR areola) NEAR/3 (sparing OR preserv* OR retain* OR retention* OR conservation* OR conserving OR graft*) AND (mastectom* OR breast-amputat* OR breast-resect* OR mammectom*))):ab,ti,kw) AND ((outcome* OR recorren* OR disease-free OR cancer-free OR surviv* OR mortalit* OR reoperation* OR complication* OR necrosis* OR necrotic* OR hematoma* OR haematoma* OR epidermolys* OR seroma* OR (Prosthesis NEAR/3 (loss)) OR ((surgical OR wound) NEAR/3 infection*) OR ((oncological) NEAR/3 (safety))):ab,ti,kw)
