# Supplementary material for: Quality of Life and Complications after Nipple- versus Skin-Sparing Mastectomy followed by Immediate Breast Reconstruction: A Systematic Review and Meta-Analysis
Source: Plast Reconstr Surg. 2023 Jun 29;152(1):12–24. doi: 10.1097/PRS.0000000000010155 (PMC10298179; doi:10.1097/PRS.0000000000010155)

Supplemental Digital Content 2. Figure - Funnel plots

Funnel plots were created to display the risk of publication bias for each domain of the Breast-Q.

Figure S1. Funnel plot for the Satisfaction With Breasts domain of the Breast-Q

Figure S2. Funnel plot for the Psychosocial Well-being domain of the Breast-Q

Figure S3. Funnel plot for the Physical Well-being domain of the Breast-Q

Figure S4. Funnel plot for the Sexual Well-being domain of the Breast-Q

Figure S5. Funnel plot for the Satisfaction With Outcome domain of the Breast-Q

Figure S1. Satisfaction With Breasts

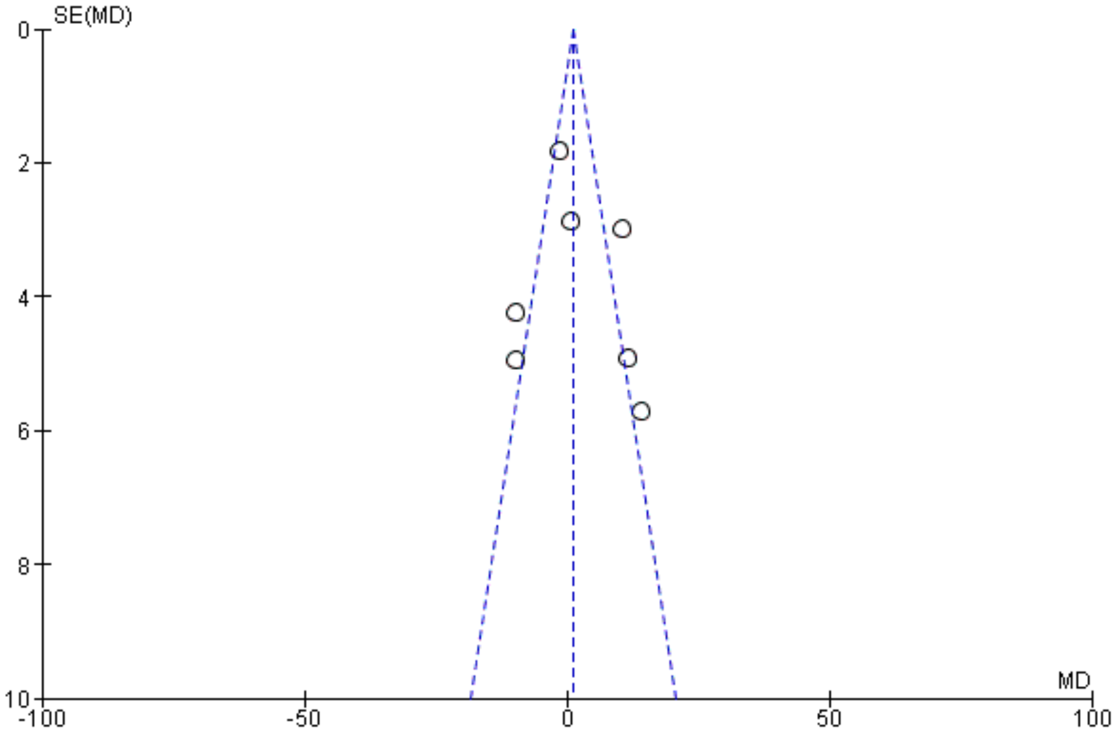

Figure S2. Psychosocial Well-being

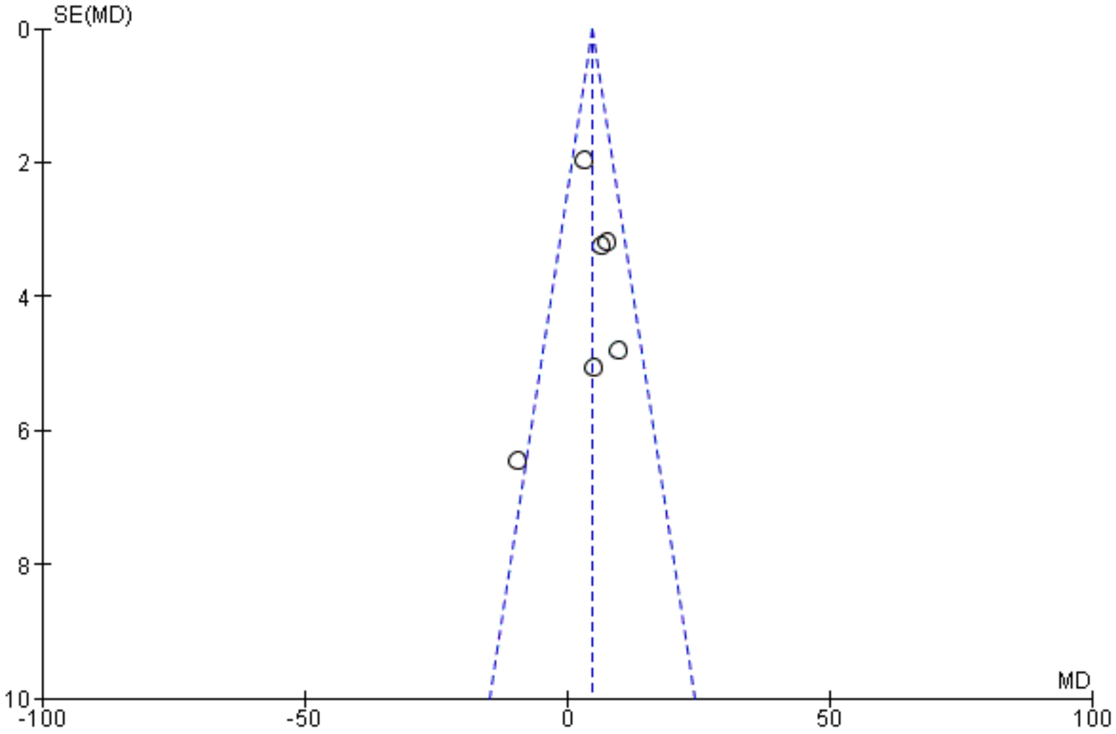

Figure S3. Physical Well-being

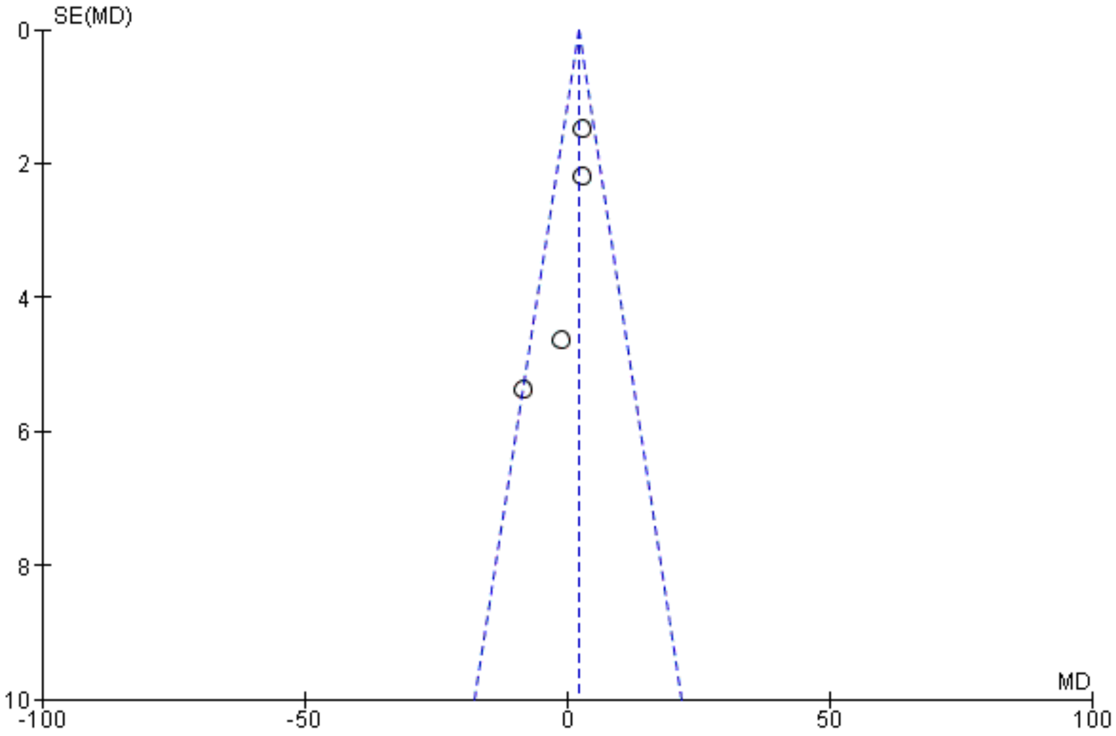

Figure S4. Sexual Well-being

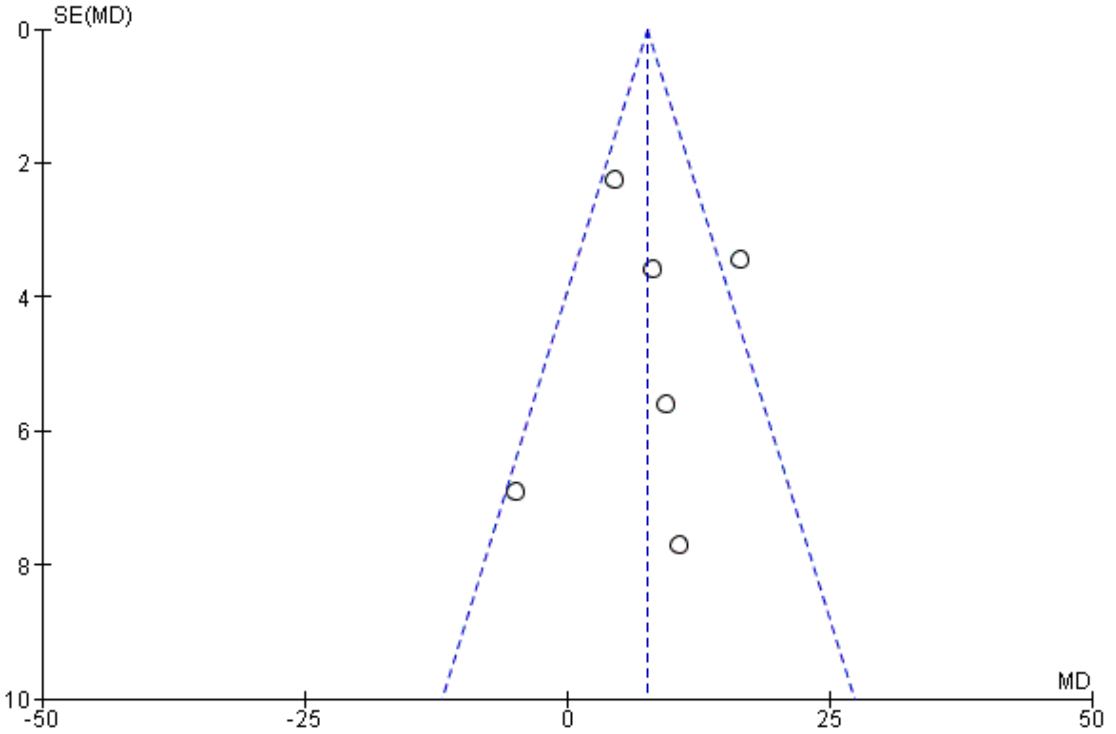

Figure S5. Satisfaction With Outcome

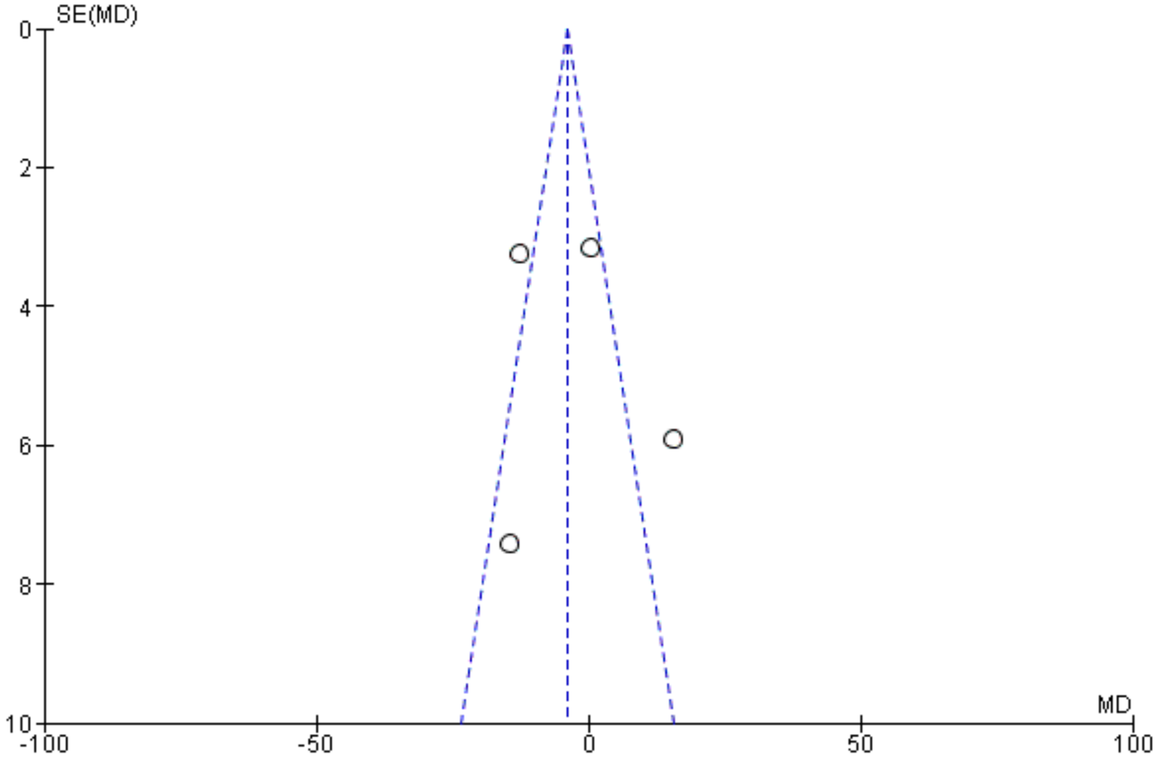

Supplement: Supplementary file 2 [file prs-152-012e-s002.pdf]
